# Supplementary material for: Much higher covariation with foveation timing by superior colliculus than primary visual cortical neuronal activity
Source: iScience. 2026 Mar 20;29(4):115432. doi: 10.1016/j.isci.2026.115432 (PMC13084408; doi:10.1016/j.isci.2026.115432)
Supplement: Document S1. Figures S1–S14 [file mmc1.pdf]

**iScience, Volume *29***

## Supplemental information

# Much higher covariation with foveation timing by superior colliculus than primary visual cortical neuronal activity

**Carlotta Trottenberg, Yue Yu, Tong Zhang, Matthias P. Baumann, Tatiana Malevich, Shweta Prasad, and Ziad M. Hafed**

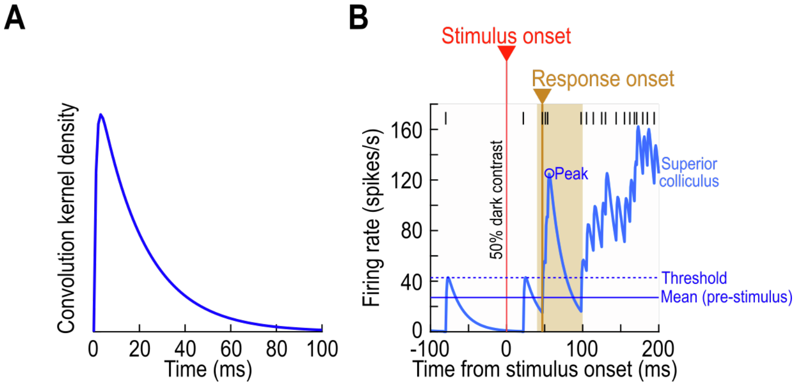


**Figure S1. Estimating trial-wise visual response onset latency from a neuron's spike times, related to Fig. 1. (A)** The convolution kernel that we employed in order to estimate firing rates from individual-trial spike times. Each spike in a trial was convolved with this kernel density function. **(B)** Example trial from an SC neuron illustrating our method for estimating trial-wise visual response onset latency. The black vertical tick marks in the top of the figure are the individual spike times emitted by the recorded neuron on the shown trial. The blue curve is the estimated firing rate curve of the trial, given the convolution of the spike times with the kernel in **A**. We estimated visual response onset latency by comparing to pre-stimulus activity. Specifically, after finding the peak firing rate (circle labeled "Peak") within a search window (brown background rectangle), we moved backwards in time until the firing rate dropped below a threshold. The threshold was defined based on measurements of pre-stimulus activity (Methods).

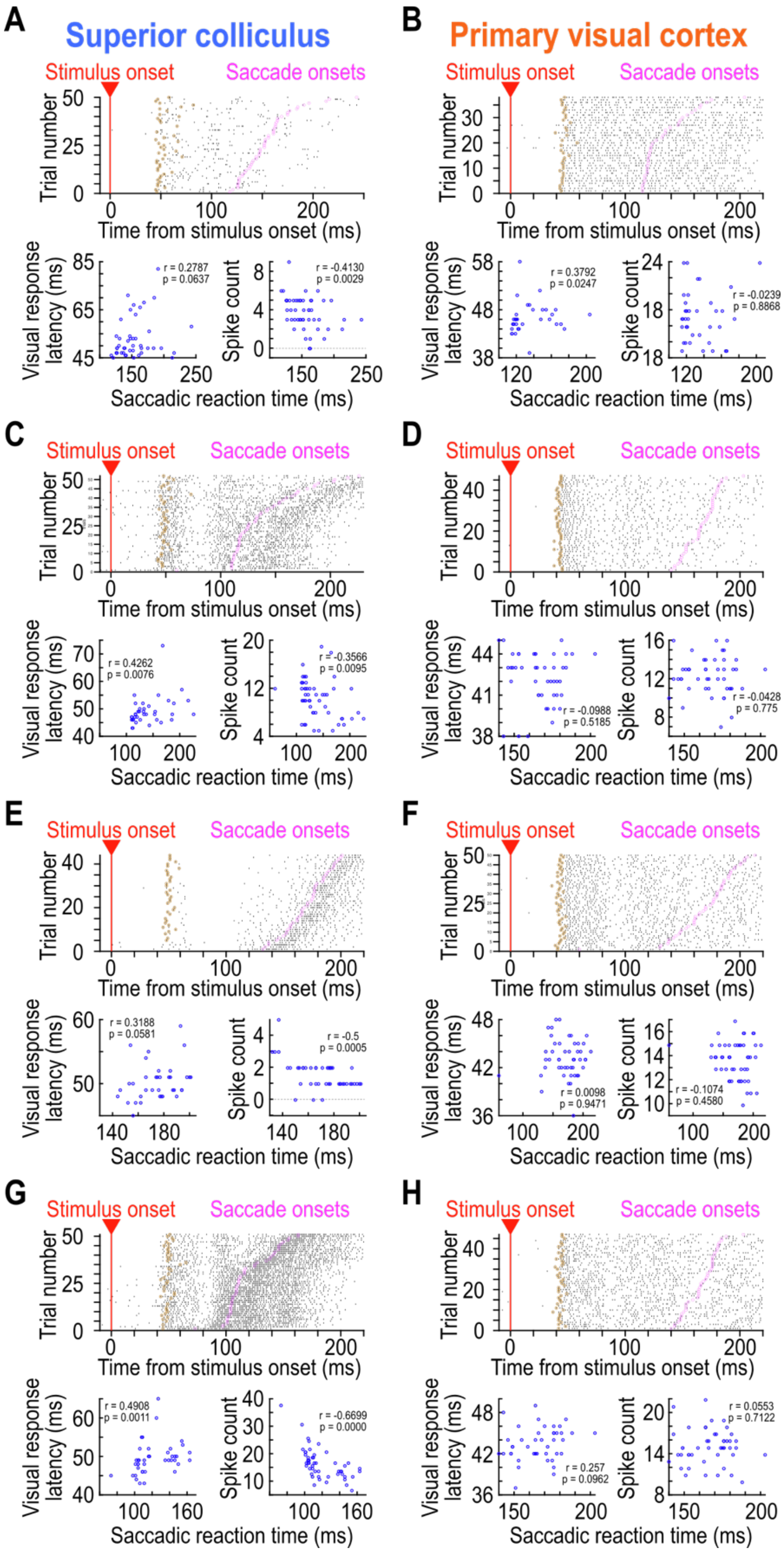


**Figure S2. Additional example neurons from both brain areas, exhibiting results that are representative of the whole population, related to Fig. 1.** This figure is formatted identically to Fig. 1, and it shows four more example SC neurons (**A**, **C**, **E**, **G**) and four more example V1 neurons (**B**, **D**, **F**, **H**). Note how the first example SC neuron (**A**) was a purely visual neuron, because it did not emit a saccade-related motor burst at the time of saccade onset. Also see Fig. 3 for the firing rate estimates of the neurons.

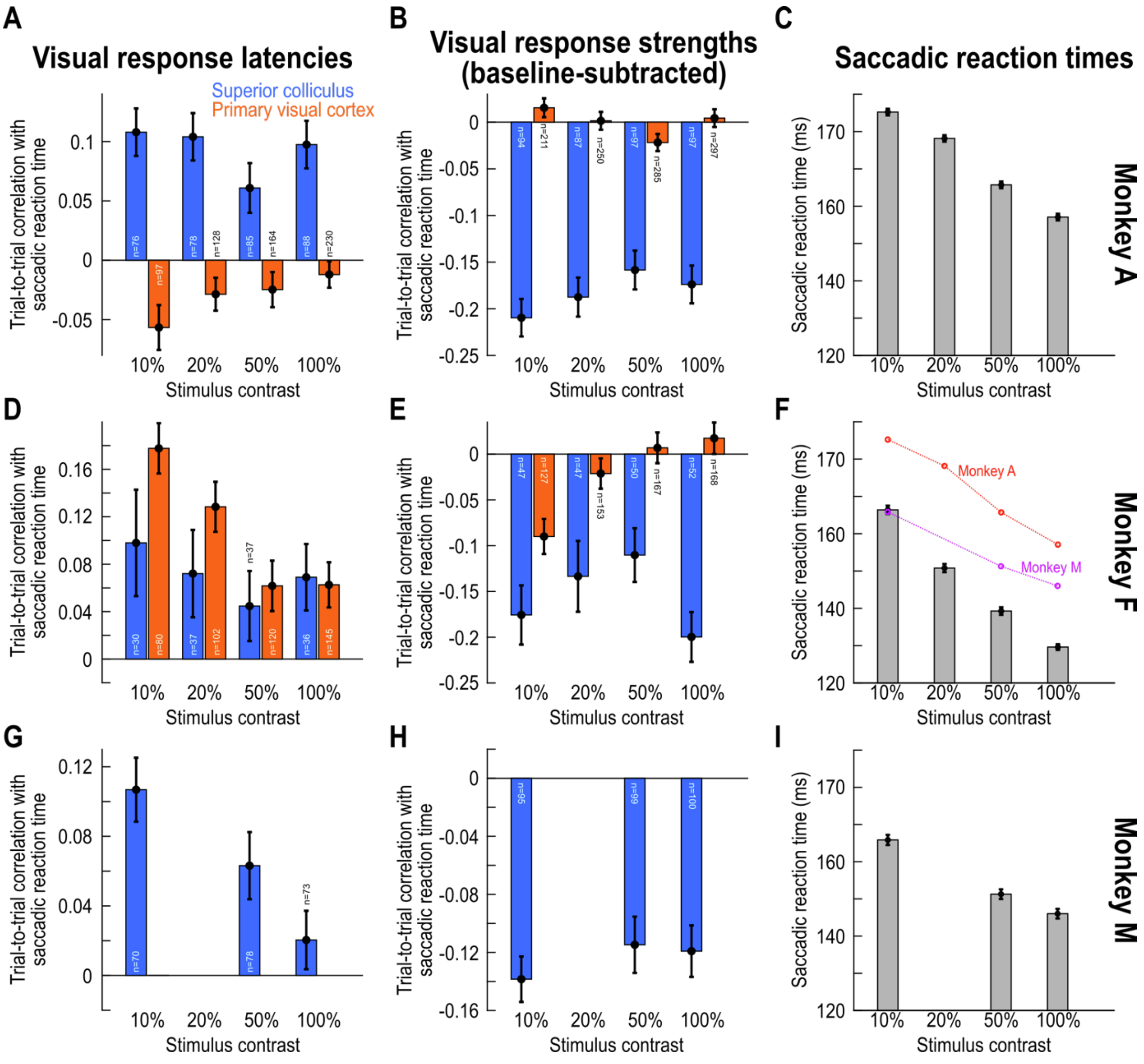


**Figure S3. Individual monkey results, related to Fig. 2. (A, B)** Similar to Fig. 2A, B, but now for only the data obtained from monkey A. Note how the results, especially for the SC, were consistent with those of Fig. 2. **(C)** The same monkey's saccadic reaction times as a function of stimulus contrast (dark stimuli shown; similar trends could be observed for bright stimuli, save for the fact that saccadic reaction times were generally slightly faster for darks than brights). Each bar shows the average saccadic reaction time across all trials from all sessions in this monkey, for a given stimulus contrast, and the error bars denote SEM. **(D-F)** Similar analyses but now for monkey F (the y-axis ranges are tailored for each monkey's results). Note how the correlation coefficients for V1 in **D** were higher than in monkey A (compare the V1 data in this panel to those in **A**). Interestingly, monkey F had much faster saccadic reaction times than the two other monkeys; for example, in **F**, we superimposed the monkey A and M saccadic reaction times of **C**, **I** in red and purple color, respectively, for easier comparison. This suggests that for more reflexive monkeys like monkey F, sensory drive from V1 (**D**) might matter more for saccadic reaction times than in other cases. In terms of the SC (blue bars), this monkey exhibited consistent results with monkey A. **(G-I)** This consistency was also clearly evident in monkey M. Note that in this monkey, we did not test 20% contrast levels (Methods). Thus, across all monkeys the SC results were highly consistent. All other conventions are similar to those in Fig. 2. Also see Fig. S5 for further classification of the SC neuron types.

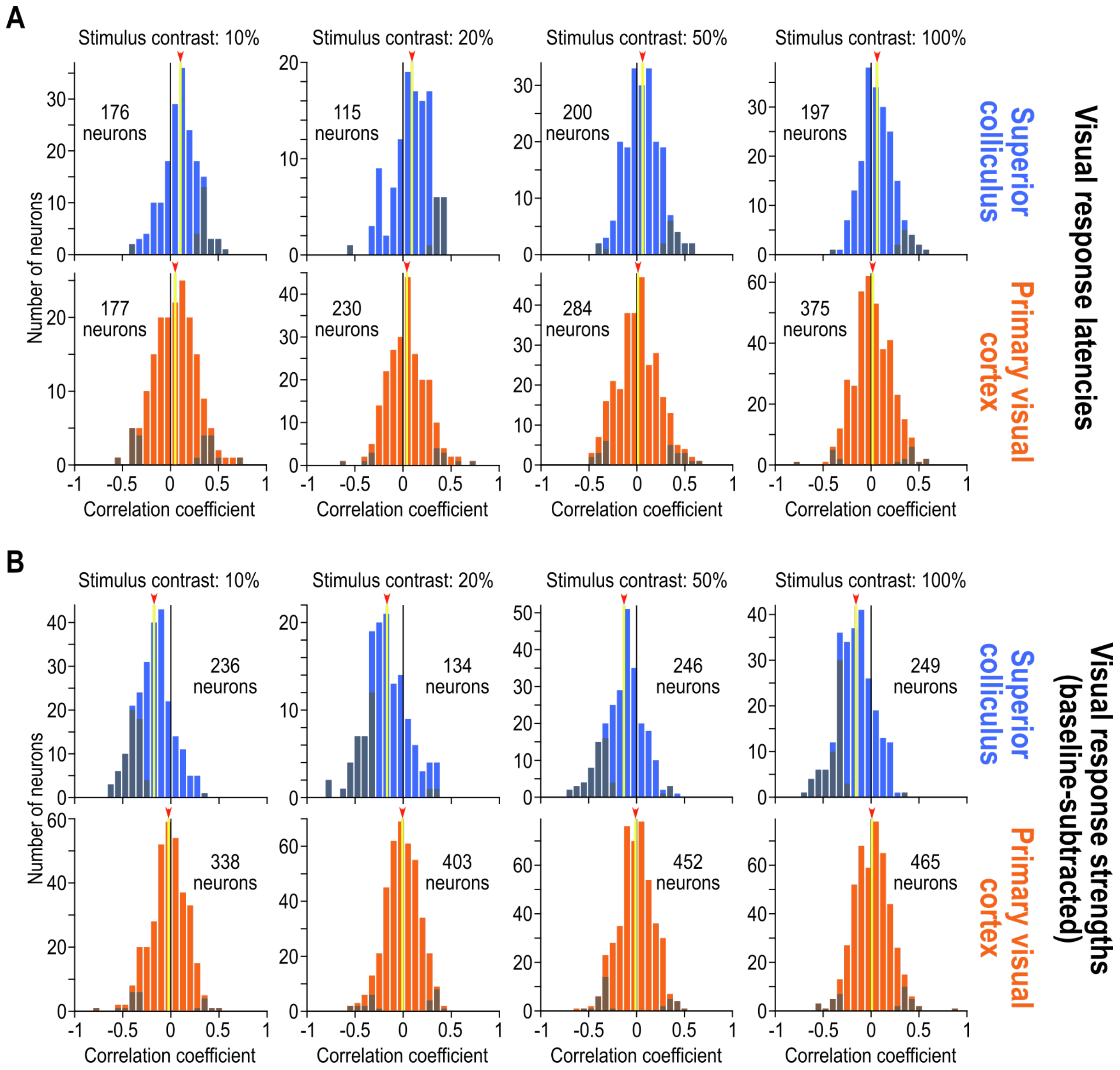


**Figure S4. Raw data underlying Fig. 2, related to Fig. 2. (A)** Raw histograms showing the underlying raw data of Fig. 2A. Each plot shows the results from one contrast level. The x-axis in each plot indicates the Spearman correlation coefficient value, and the y-axis shows the number of neurons exhibiting this correlation coefficient value. The yellow vertical line in each plot (and the red arrowhead on its top) shows the mean of the shown population, and the dark bars indicate the neurons that individually had significant Spearman correlation coefficient values. Note how the SC population had consistently positive correlation coefficients, whereas the V1 population had a mean correlation coefficient very close to zero. **(B)** Raw histograms showing the underlying raw data of Fig. 2B. Note how the SC results were even stronger than in **A**, but in the negative direction, consistent with Fig. 2. Moreover, the V1 distributions were all centered around zero, again consistent with Fig. 2. Also see Fig. S6 for further delineation of the SC data as a function of whether the neurons were visual or visual-motor neurons.

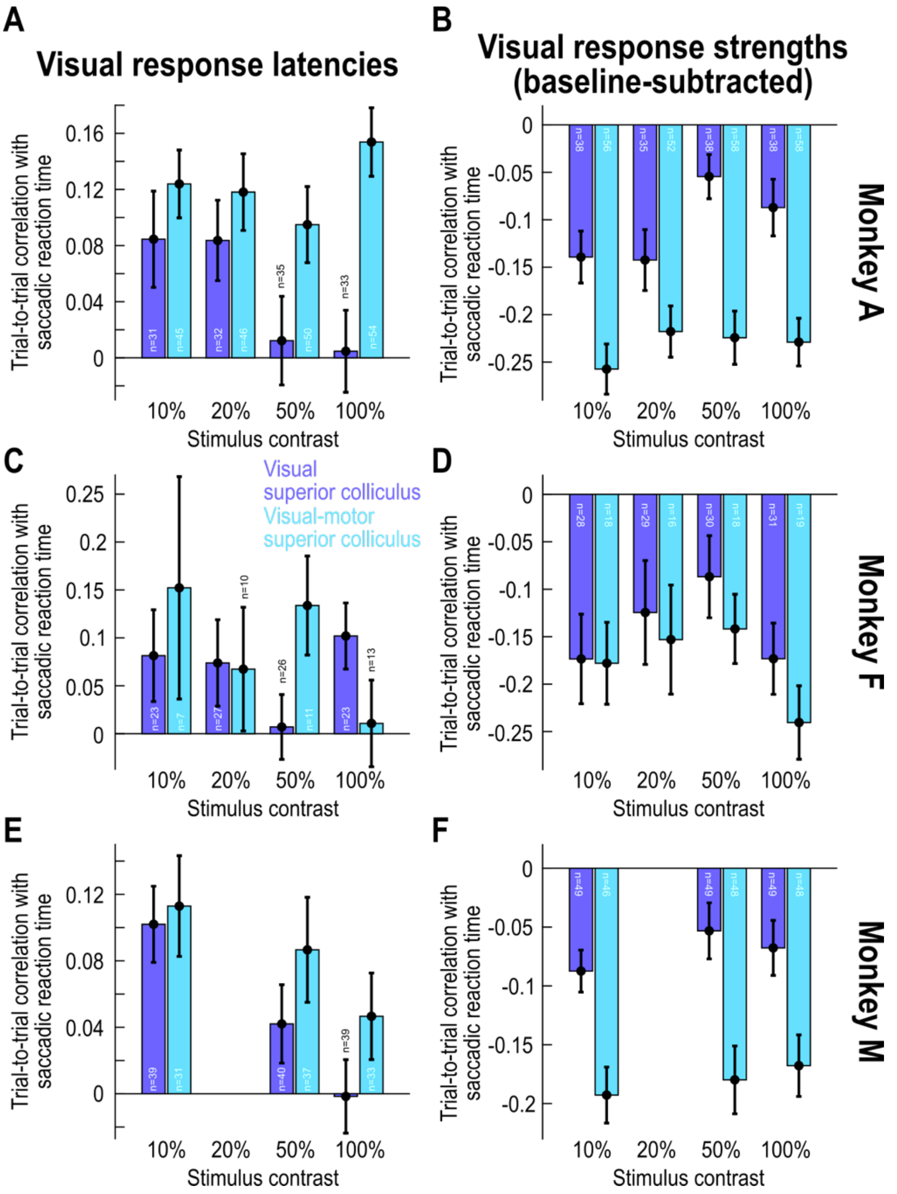


**Figure S5. Individual monkey results for Fig. 4, related to Fig. 4.** This figure is formatted similarly to Fig. S3 above, but now separating the SC visual and visual-motor neurons. All monkeys showed consistent results. The figure is formatted identically to Fig. 4, except that we do not show the V1 data, which are shown in Fig. S3.

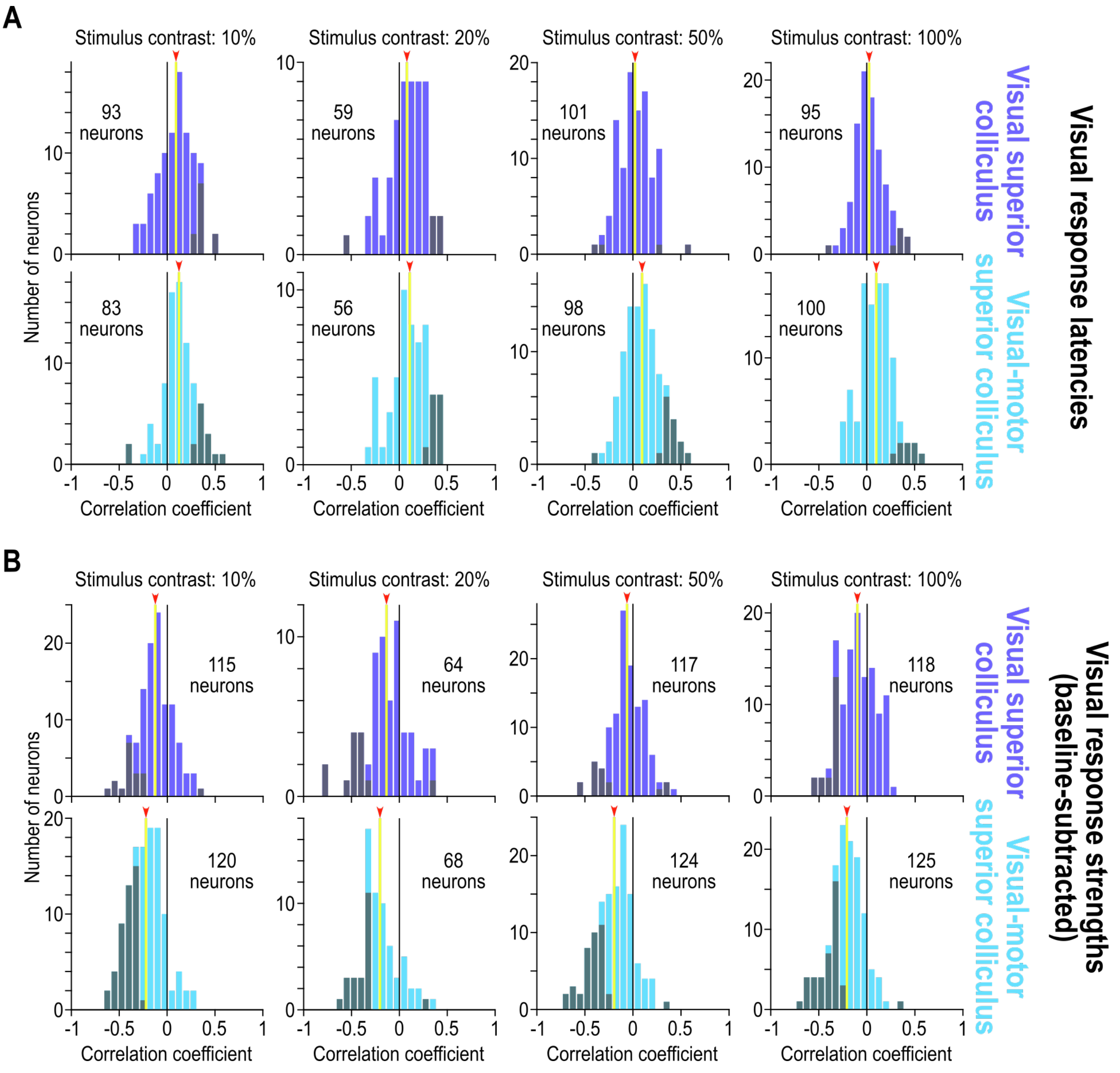


**Figure S6. Raw data underlying Fig. 4, related to Fig. 4. (A)** Similar to Fig. S4A, but now comparing SC visual (top row) to SC visual-motor (bottom row) neurons. Note how both the visual and visual-motor neurons showed positive correlation coefficient values across the population (yellow vertical lines indicating the means), and also note how the effects were stronger for the visual-motor neurons (compare the yellow lines), as summarized in Fig. 4A. **(B)** The effects were even stronger for visual response strengths in the SC, but in the negative direction, again as summarized in Fig. 4B. All other conventions are similar to those in Fig. S4.

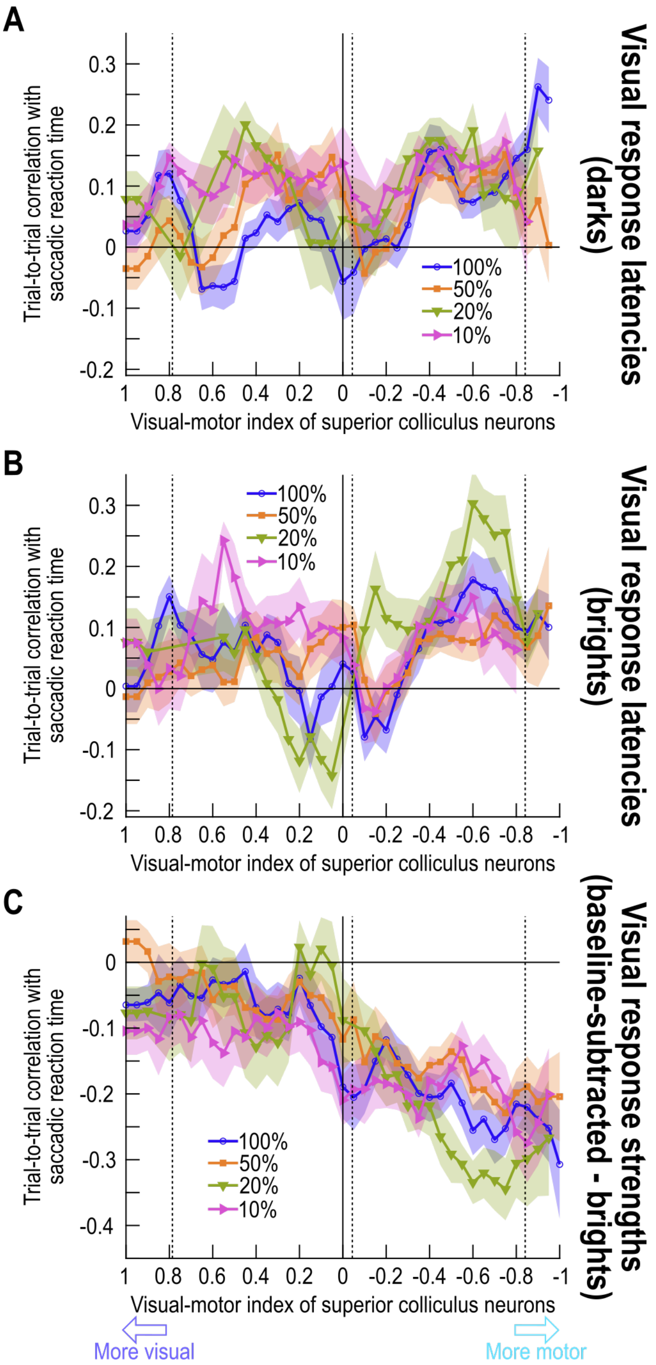


**Figure S7. Dependence of Spearman correlation coefficient values on functional depth within the SC, as assessed via visual-motor indices, related to Fig. 5. (A)** Similar to Fig. 5, but now plotting the Spearman correlation coefficients (relating SC visual response latency, as opposed to visual response strength in Fig. 5, to saccadic reaction time) as a function of the visual-motor index (VMI) of the recorded SC neurons. More visual VMI's (left side of the x-axis) are associated with more superficial SC neurons, and more negative VMI's (right side of the x-axis) are associated with more deep SC neurons. The correlation coefficients increased with increasing functional depth within the SC, consistent with the results of Fig. 4. Note that the increase with the shift of VMI's towards more motor values (rightward on the x-axis) was generally weaker than the effects that we observed with visual response strengths in Fig. 5. This is consistent with our earlier observations that visual response strength, not latency, was the better predictor of saccadic reaction times in the SC data (Figs. 2, 4). **(B)** Same as **A** but for bright visual stimuli. Similar trends were observed. **(C)** Same as Fig. 5 but for bright stimuli. Similar trends were observed. Error bars denote SEM.

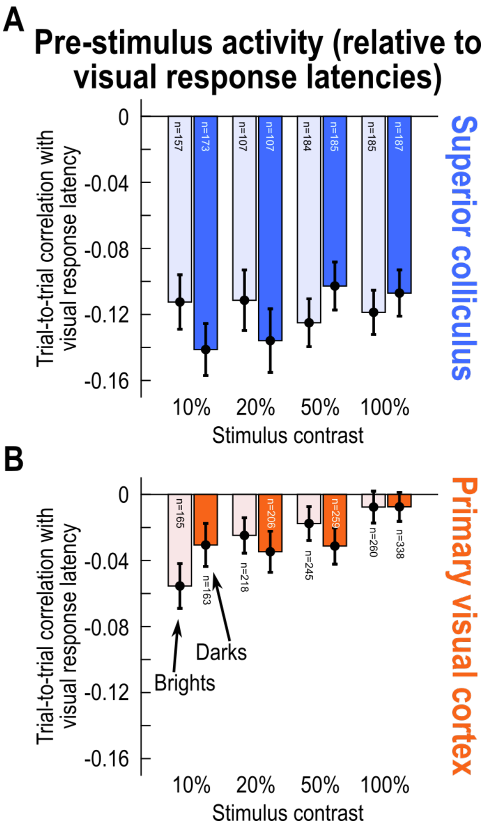


**Figure S8. Relating trial-by-trial variability in pre-stimulus (baseline) activity to trial-by-trial variability in visual response latency, in both the SC and V1, related to Fig. 8. (A)** For each SC neuron and stimulus condition, we measured trial-by-trial pre-stimulus activity and correlated it with trial-by-trial visual response onset latency. Negative correlations mean weaker pre-stimulus activity for later stimulus-evoked visual response onset latency. The results indicate that visual response onset latencies were a function of pre-stimulus SC state, and not completely independent of it. More importantly, there was no difference in the effects between dark and bright contrasts (U=418557, z=0.14, p=0.8885, $n_{brights}$=1067, $n_{darks}$=689; Mann-Whitney U test comparing darks and brights across contrasts). This suggests that the weaker correlations to behavior (rather than visual response onset latency) in pre-stimulus activity for bright contrasts (Fig. 8C) was mediated by a mechanism other than a link between pre-stimulus activity and visual response latency. Potentially, this other mechanism could include visual response strength correlations (Figs. 8B, S9) because our visual response strength analyses were always independent of pre-stimulus activity (Methods). **(B)** For V1, the visual response latencies for low contrast stimuli (except for 20% brights) did generally depend on pre-stimulus activity (10% brights: W=2962, z=-3.90, p<0.0001, n=146; 10% darks: W=3547, z=-2.41, p=0.0158, n=150; 20% brights: W=7040, z=-1.92, p=0.0548, n=196; 20% darks: W=5295, z=-2.64, p=0.0084, n=190; Wilcoxon signed-rank test against zero). This could partially explain why V1 visual response latencies were better correlated to saccade timing with low contrast bright stimuli (Fig. 8D). Error bars denote SEM, and the numbers of neurons included in each analysis are included in the figure.

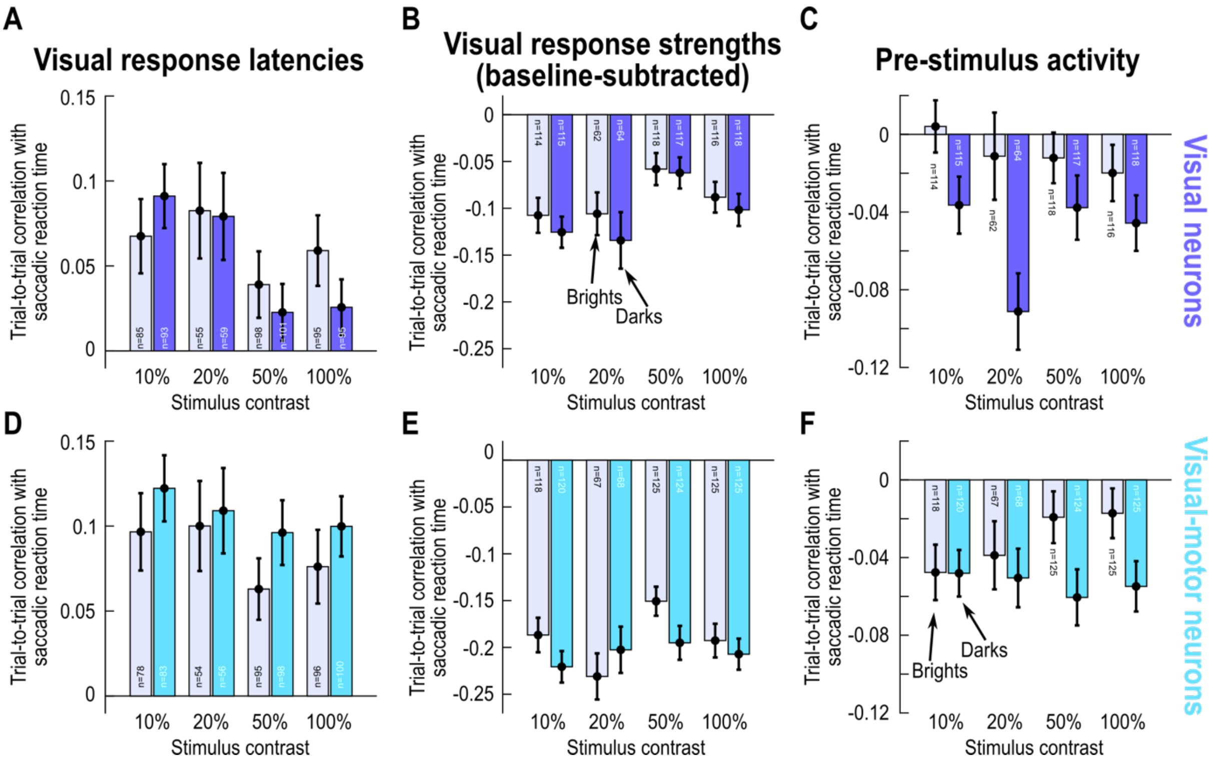


**Figure S9. Similar dependencies on dark versus bright contrasts in both SC visual and visual-motor neurons, related to Fig. 8. (A-C)** Same analyses as in Fig. 8A-C but only for the visual-only neurons of our SC population. Saturated bars show results for dark contrasts, and faint bars show results for bright contrasts. In terms of visual response onset latency (**A**), there was no statistically significant difference between dark and bright contrasts (U=116511, z=-0.84, p=0.4007, $n_{brights}$=333, $n_{darks}$=348, Mann-Whitney U test comparing brights and darks across contrast levels). This was also true for visual response strength (**B**) (U=168217, z=-0.75, p=0.4541, $n_{brights}$=410, $n_{darks}$=414; Mann-Whitney U test comparing brights and darks across contrast levels). However, for pre-stimulus activity (**C**), there were weaker correlations with trial-by-trial saccadic reaction times in the case of bright versus dark stimuli (U=159130, z=-3.41, p=0.0006, $n_{brights}$=410, $n_{darks}$=414), but only 20% contrast was individually significant after Bonferroni correction. **(D-F)** Same analyses as in Fig. 8D-F but only for the visual-motor SC neurons of our population. For visual response strength (**E**) (U=183437, z=-1.97, p=0.0493, $n_{brights}$=435, $n_{darks}$=437) and pre-stimulus activity (**F**) (U=181828, z=-2.40, p=0.0163, $n_{brights}$=435, $n_{darks}$=437), there were weaker effects in the case of bright contrasts; however, none of the individual contrast comparisons reached significance after Bonferroni correction. Thus, the generally weaker effects for bright contrasts in Fig. 8 were consistent across SC neuron types.

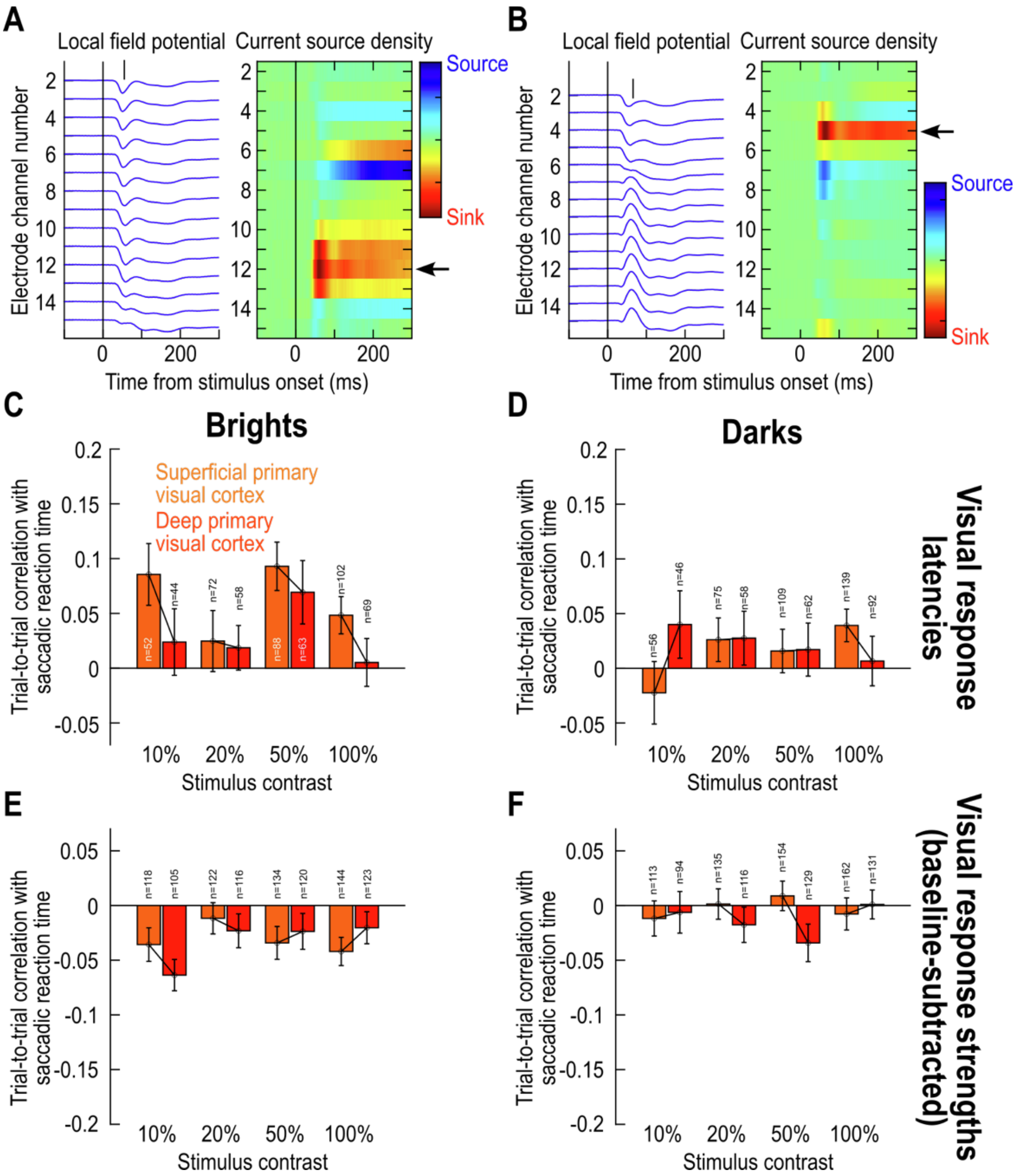


**Figure S10. No consistent difference in effects between superficial and deep V1 layers, related to Fig. 8. (A)** Using current source density (CSD) analysis (Methods), we classified our V1 neurons according to whether they were in putative superficial or deep V1 layers. In the left panel, we plotted stimulus-evoked local field potential (LFP) deflections as a function of time from stimulus onset; each trace shows the deflection from one channel of the recording electrode array from one example session. In the right panel, we used the CSD pattern inferred from the LFP deflections of the left panel, and we identified the sink at channel 12 (highlighted with a black arrow); note how this was also the channel with the earliest stimulus-evoked responses. Thus, for this session, channel 12 corresponded to the putative input layer: we classified neurons above this channel as superficial V1 neurons, and neurons below as deep V1 neurons (Methods). **(B)** In another example session, the putative input layer was identified at channel 5 of the electrode array. Thus, neurons from channels above channel 5 were the superficial neurons, and neurons from channels below were the deeper neurons. **(C-F)** Across all sessions, after alignments like in **A**, **B**, we repeated our analyses but only for subsets of V1 neurons. There were no systematic differences in effects as a function of V1 depth. Only in the case of bright stimuli (**C**) were there larger correlations in the superficial neurons (U=89797, z=1.97, p=0.0494, $n_{superficial}$=314, $n_{deep}$=234; Mann-Whitney U test across contrasts). Note that we did not analyze effects related to pre-stimulus activity as a function of V1 depth because such activity was very low anyway, and also because it did not strongly depend on such depth (see Fig. S11).

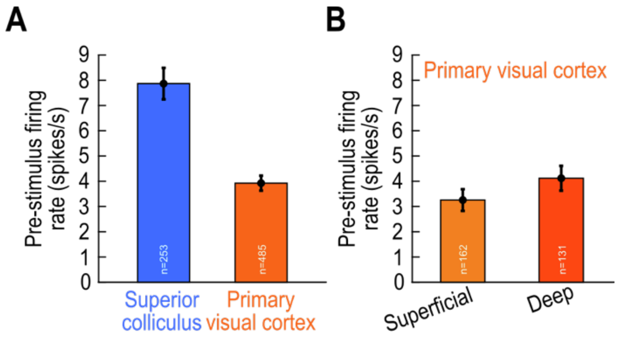


**Figure S11. Weaker pre-stimulus (baseline) activity in V1 than in the SC, related to Fig. 8. (A)** SC neurons possessed twice the pre-stimulus (baseline) firing rate of V1 neurons. **(B)** Neurons in the superficial and deep V1 layers had similar pre-stimulus firing rates. The input layers of V1 had even weaker pre-stimulus activity (1.44 spikes/s +/- 0.395 spikes/s SEM; n=38 neurons). Error bars denote SEM, and the numbers of neurons in each analysis are shown in the figure.

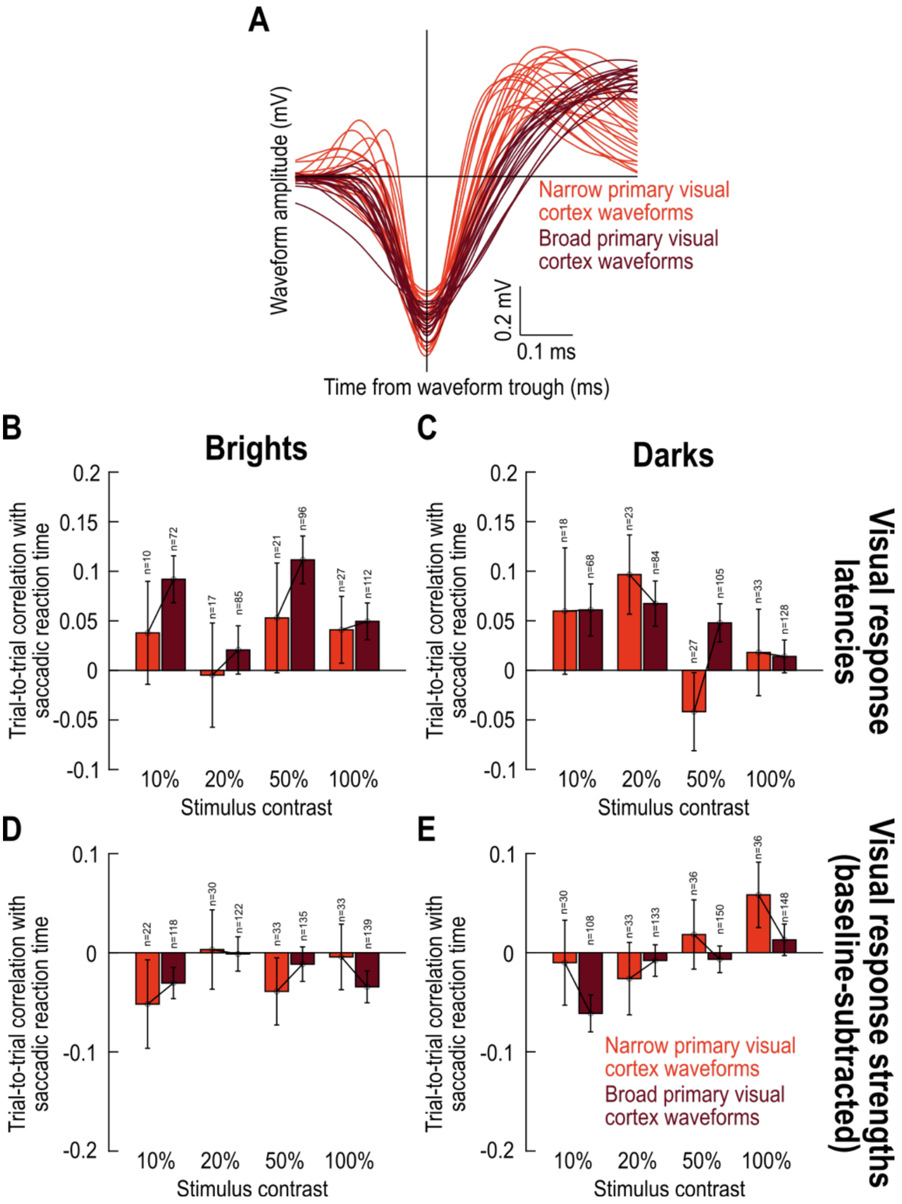


**Figure S12. No consistent difference in effects between narrow- and broad-spiking V1 neurons, related to Fig. 8.** (**A**) Twenty randomly chosen neurons having a broad action potential waveform (dark color), and twenty having a narrow action potential waveform (lighter color; Methods). **(B-E)** None of our analyses revealed any significant differences between narrow- and wide-spiking neurons in V1, in terms of correlations to behavioral variability.

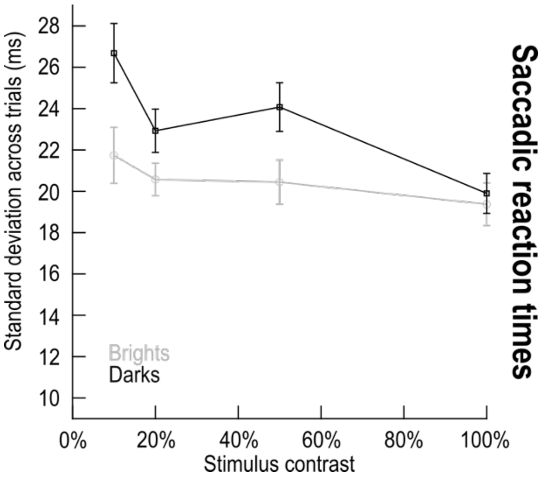


**Figure S13. High variability in foveation timing, related to Fig. 10.** For each behavioral session, we measured the standard deviation of saccadic reaction times across trials. The shown summaries are averages and SEM ranges across sessions (dark and square symbols indicate dark contrasts, and bright and circle symbols indicate bright contrasts). In comparison to Fig. 10, the timing variability in behavior was much higher than the timing behavior of visual response onset latencies in both the SC and V1.

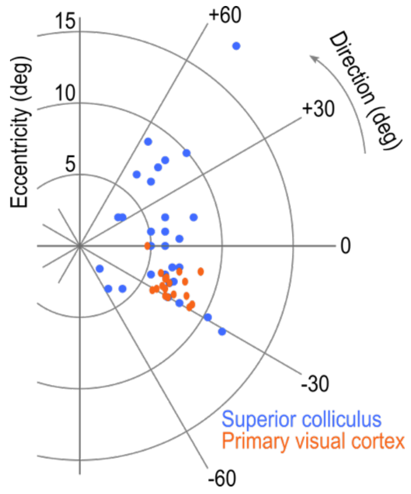


**Figure S14. Sampled eccentricities and direction in SC and V1, related to Fig. 2.** This figure shows the saccade target locations used across sessions in the SC (blue circles) and V1 (orange ovals). Such locations were chosen according to the average receptive field location in each electrode penetration (Methods). In both brain areas, we had similar ranges of eccentricities of the saccade targets. In the SC, we sampled both upper and lower visual field stimulus locations.
